# Exploring the relationship between environmental DNA concentration and biomass in Asian giant softshell turtle (*Pelochelys cantorii*)

**DOI:** 10.7717/peerj.16218

**Published:** 2023-10-04

**Authors:** Xiaoyou Hong, Kaikuo Wang, Liqin Ji, Xiaoli Liu, Lingyun Yu, Jie Wei, Yakun Wang, Chengqing Wei, Wei Li, Xinping Zhu

**Affiliations:** 1 Key Laboratory of Tropical and Subtropical Fishery Resources Application and Cultivation, Ministry of Agriculture and Rural Affairs, Pearl River Fisheries Research Institute, Chinese Academy of Fishery Sciences, Guangzhou, China; 2 National Demonstration Center for Experimental Fisheries Science Education, Shanghai Ocean University, Shanghai, China

**Keywords:** Environmental DNA, *Pelochelys cantorii*, ND5, Quantitative PCR, Biomass

## Abstract

In recent years, environmental DNA (eDNA) technology has become an accepted approach for investigating rare and endangered species because of its economic efficiency, high sensitivity, and non-invasiveness. The Asian giant softshell turtle (*Pelochelys cantorii*) is a first-class protected aquatic animal in China, and traditional resource survey methods have not identified its natural populations for many years. In this study, primers and a TaqMan probe targeting *ND5* were designed, reaction conditions were optimized, a standard curve was constructed using synthetic DNA, and an eDNA quantitative PCR (qPCR) detection method was established. The eDNA detection technology for *P. cantorii* revealed that the number of species in the experimental pools showed a significant linear relationship with the eDNA concentration (*p* < 0.05). The eDNA concentration was negatively correlated with the length of time after the removal of *P. cantorii* and retention in the water body for 9 days. The qPCR detection method for *P. cantorii* eDNA established in this study can be applied to the qualitative detection of *P. cantorii* in water bodies, as well as to preliminary evaluation of its relative biomass. This can serve as a baseline for the investigation of natural *P. cantorii* population and the evaluation of its wild release effects.

## Introduction

Understanding species distribution is essential for ecosystem protection, particularly for monitoring endangered species (Begon, Townsend & Harper, 2005). Environmental DNA (eDNA) has recently gained popularity as a research topic and is now widely used in ecological monitoring worldwide (Rees et al., 2015). Since Ficetola et al. (2008) successfully detected the invasive North American bullfrog (*Rana catesbeiana*) in freshwater rivers using eDNA from water samples, an increasing number of studies have used eDNA extracted from water bodies to investigate and monitor fish, reptiles, and amphibians for species detection, biomass estimation, and biodiversity (Thomsen & Willerslev, 2015; Zaiko et al., 2018; Wang et al., 2019).

The use of eDNA has yielded promising results in the monitoring of endangered animals. Goldberg et al. (2011) successfully performed PCR amplification of 85 and 78 bp fragments of the mitochondrial cytochrome b gene region of two rare species, the tailed frog (*Ascaphus montanus*) and the Idaho giant salamander (*Dicamptodon aterrimus*), respectively, in river water samples with five different species densities. Wilcox et al. (2013) successfully detected two rare species, brook trout (*Salvelinus fontinalis*) and bull trout (*Salvelinus confluentus*), in rivers using eDNA combined with quantitative PCR (qPCR). Piaggio et al. (2014) collected water samples from six field sites, five of which tested positive for Burmese python (*Python bivittatus*) eDNA, aligning with their field distribution. Harper et al. (2019) effectively utilized eDNA to monitor the threatened crucian carp (*Carassius carassius*). Schmidt et al. (2021) detected traces of endangered freshwater mussels (*Margaritifera margaritifera*) in stream samples using eDNA techniques, and found that eDNA sampling yielded higher detection rates and lower costs compared to conventional methods. Additionally, using eDNA technology, a baleen whale that appeared in a coastal bay was identified as belonging to the Bryde’s whale coastal subspecies (Zhang et al., 2023).

In addition, eDNA technology has been successful in monitoring turtle resources. Davy, Kidd & Wilson (2015) successfully detected seven native freshwater turtles and one exotic turtle in the same area, using environmental DNA. Using species-specific qPCR assays, flattened musk turtles *(Sternotherus depressus*) were found to have a higher probability of eDNA detection during the warm season, and upon which an occupancy rate model was established (Souza et al., 2016). The eDNA-based methodology can detect the presence of the European pond turtle (*Emys orbicularis*), even at low density, with better accuracy than visual observation (Raemy & Ursenbacher, 2018). Feist et al. (2018) developed an eDNA method for detection of a federally endangered species alligator snapping turtles (*Macrochelys temminckii*). eDNA surveys help to identify winter hibernacula of the northern map turtle (*Graptemys geographica*) (Feng, Bulté & Lougheed, 2019). eDNA confirmed extant populations of the cryptic Irwin’s turtle (*Elseya irwini*) within its historical range, where the species had not been formally recorded for >25 years (Villacorta-Rath et al., 2022).

The distribution of species can be effectively detected through eDNA, and some results regarding species biomass have been obtained using qPCR of eDNA. For example, Takahara et al. (2012) and others showed a significant linear relationship between the concentration of carp (*Cyprinus carpio*) eDNA per liter and biomass through using qPCR analysis in laboratory and field pond experiments. Pilliod et al. (2013) found a positive correlation between the environmental concentration of tailed frogs (*Ascaphus montanus*) and giant salamanders (*Dicamptodon aterrimus*) in rivers and their biological densities. Klymus et al. (2015) conducted laboratory pool research and found a significant positive correlation between the eDNAconcentration of invasive bream and biomass, which was not affected by environmental temperature. The biomass of ayu (*Plecoglossus altivelis*) in rivers can be effectively detected using eDNA (Doi et al., 2017). Further, eDNA concentration can effectively reflect spatiotemporal abundance changes in sockeye salmon (*Oncorhynchus nerka*) during the spawning season (Tillotson et al., 2018).

**Figure 1:**
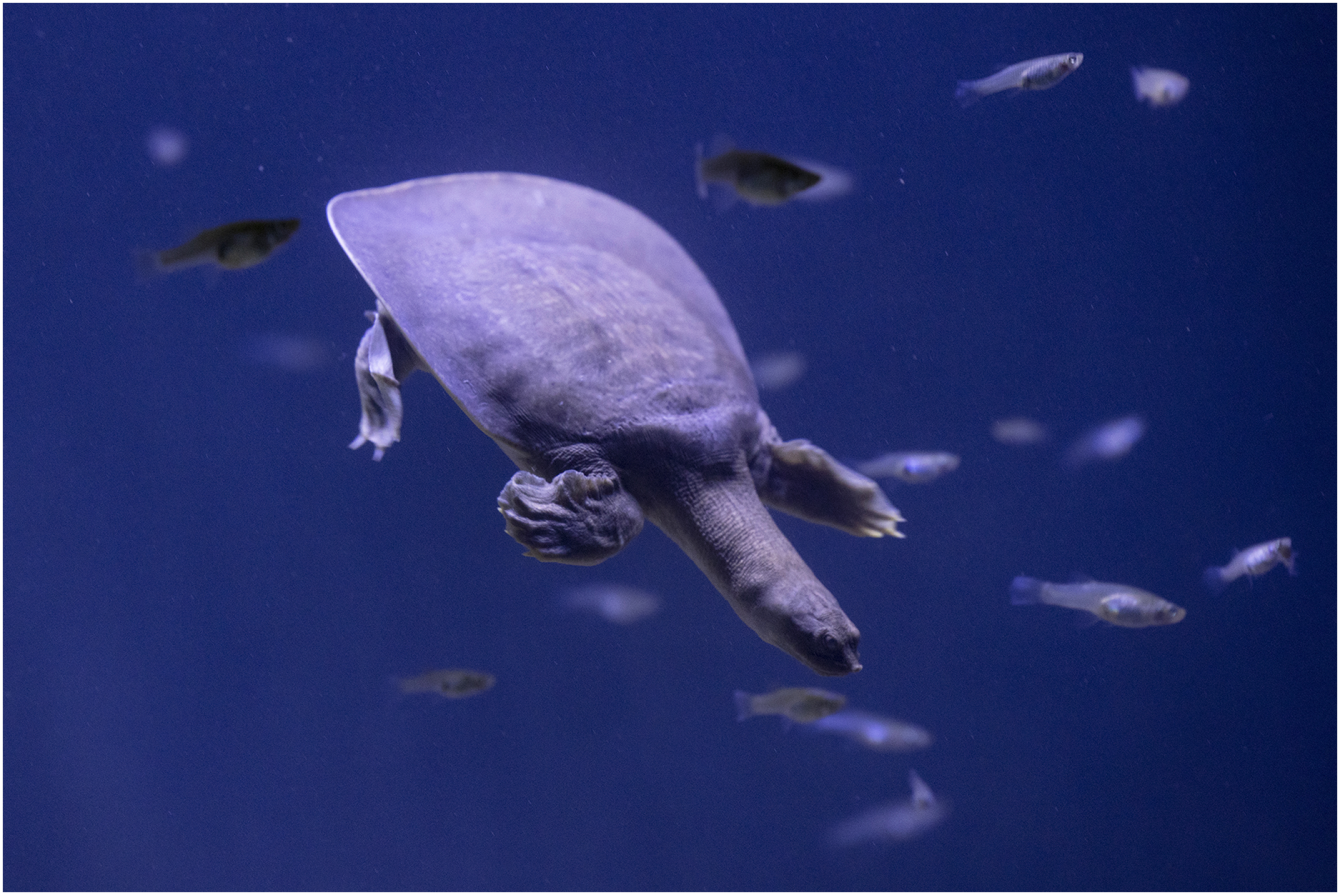
Asian giant softshell turtle (*Pelochelys cantorii*).

In China, 36 known species of turtles and tortoises are threatened by habitat change and human activity (Zhou, 1998). One of the largest inland aquatic turtles in China is the Asian giant softshell turtle (Fig. 1), which is found in the Yangtze River Valley in southern China and some Southeast Asian countries (Shi, 2011). This species is similar to the functionally extinct Yangtze giant softshell turtle (*Rafetus swinhoei*) in size and habitat (Ren et al., 2022). Since the 1970s, its distribution area has decreased, and its population has declined owing to habitat deterioration and poaching; it has been listed as a national-level aquatic natural wild animal in China (Yuan et al., 2001). Research on turtles has mainly focused on breeding (Zhu et al., 2015) and genetic biology (Xie et al., 2022) of artificially preserved individuals. There are only 13 known natural *P. cantorii* individuals in China (Hong et al., 2019), but four have been successfully bred with nearly 1,000 offspring (Hong et al., 2022). Despite the establishment of many nature reserves for the *P. cantorii* in China, no individuals have been detected for many years. Traditional resource investigation methods, such as direct observation, trapping, tagging, and surveying tracks or other signs of animal presence, are unable to meet the requirements for investigating this endangered animal under field conditions, as *P. cantorii* naturally spends long time periods covered in sand. More informative scientific technologies are urgently needed to improve the investigation of turtle resources. The eDNA of aquatic organisms is DNA that is released into the environment through biological processes, such as the shedding of skin, scales, or nails, and the excretion of feces or urine (Thomsen et al., 2012). Vietnam successfully used eDNA technology to detect the presence of the fourth *R. swinhoei* species in the world (Asianturtleprogram.org, 2018). Therefore, this study aimed to establish and optimize a turtle eDNA fluorescence qPCR detection system to provide a better method for monitoring natural populations of turtles. This will assist traditional resource investigation methods to improve accuracy of species identification and distribution and reduce the time and labor required for data and sample collection. Providing a more informative scientific method for protecting endangered aquatic animals such as turtles has important practical significance and good application prospects.

## Materials and Methods

### Asian giant softshell turtle-specific primers and TaqMan probe

Based on the mitochondrial genome sequence of *P. cantorii* from Foshan City, Guangdong Province (GenBank: KT962834.1), real-time fluorescent qPCR primers (forward and reverse) and probe were designed using Primer Express software (version 3.0), and a group of primers and TaqMan probes were selected for the *ND5* gene in the *P. cantorii* mitochondrial genome (Table 1). Primers and probes were synthesized by Sangon Biotech Co., Ltd., (Shanghai, China). The 5′ end of the TaqMan probe was labeled with 6-FAM, and the 3′ end was modified with BHQ-1. The total length of the amplicon, including the primers, was 115 bp. The *ND5* primer sequence was matched to the National Center for Biotechnology Information (NCBI) nucleotide database and primer BLAST was used to evaluate primer specificity. The target regions of all available *P. cantorii* mitochondrial genome data from the NCBI database (GenBank accession numbers: KT962834.1; Zhaoqing, Guangdong, GenBank: OQ569929; Chaozhou, Guangdong, GenBank: OQ450313; Qinzhou, Guangxi, GenBank: OQ569930; Xiamen, Fujian, GenBank: JN016746.1; Hangzhou, Zhejiang, GenBank: OQ569931; Wenzhou, Zhejiang, GenBank: JN016747.1) were compared to verify similarity.

**Table 1:** *ND5* primers and probe.

Name	Type	Primer/Probe sequence (5′-3′)	Length (bp)
*ND5*-F	Forward primer	CATCCCACACAAACGCCTGA	20
*ND5*-R	Reverse primer	GGTTGGAATCGTGGTGGTCC	20
*ND5*-P	Probe	6-FAM-ACCGCAACATCCCTTACCGCAGCCT-BHQ-1	25

Artificially bred *P. cantorii* that died of illness and the tissues of three further related species (spiny softshell turtle, *Apalone spinifera*; Chinese softshell turtle, *Pelodiscus sinensis*; Burmese narrow-headed softshell turtle, *Chitra vandijki*) were used for DNA extraction using the MicroElute Genomic DNA Kit (OMEGA, Biel/Bienne, Switzerland), following the manufacturer’s instructions. All samples were obtained from artificially cultured individuals and all tissue samples were collected from the skirt muscle. Before performing qPCR amplification, the DNA of each species was diluted to 20 ng/μl and then amplified. The DNA of Asian giant softshell turtles and the other three softshell turtles were extracted as templates for real-time fluorescence qPCR to verify the effectiveness of the primers and probe. The real-time qPCR amplification system was as follows: 10 μl of 2× Premix ex Taq TM (probe qPCR), 0.4 μl of forward and reverse primers, 0.8 μl of TaqMan probe, 0.2 μl of Rox II, and 6.2 μl of sterile water. The total volume was 20 μl.

### Reaction conditions for real-time qPCR

#### qPCR annealing temperature test

The annealing temperatures of the newly designed primers and probe (60 °C, 63 °C, and 65 °C) were tested using *P. cantorii* DNA. Each DNA sample was tested three times at each temperature. The two-step qPCR amplification standard process was as follows: first, pre-denaturation at 95 °C for 30 s; second, PCR reaction, 95 °C, 5 s, 60 °C/63 °C/65 °C, 34 s. Nuclease-free water was added as a negative control to detect contamination.

#### qPCR condition optimization

After determining the optimal annealing temperature, we determined the optimal qPCR efficiency by combining eight pairs of primers and probe concentrations (Table 2).

**Table 2:** Primer-probe concentration combination.

Groups	*ND5* primer concentration (µM)	*ND5* probe concentration (µM)
1	0.5	2
2	0.5	5
3	1	0.5
4	1	1
5	1	2
6	1	5
7	2	1
8	3	3

### Standard curve preparation

The target fragment sequence was synthesized into a standard substance by Sangon Biotech Co., Ltd., (Shanghai, China) for the initial dilution, and the DNA concentration was measured using a spectrophotometer. According to the calculation formula for the DNA copy number: $\eqalign{{\rm copy\; number\; (copies/\mu L)\; = }\textstyle{{{\rm Avogadro\; constant\; (6}{\rm .02\; \times \; 1,023)\; \times \; DNA\; concentration\; ng/\mu L\; \times 10 _{- 9}}} \over {{\rm DNA\; length\; bp\; \times\, 660}}}{\rm \; }}$ (Wei, Wang & Zhang, 2020), the standard substance was diluted to 10^1^–10^10^ copies/µL. Amplification was conducted according to the qPCR experimental steps described above, a standard curve was drawn, and sensitivity was determined.

### Stability of eDNA concentration and degradation time of the Asian giant softshell turtle

The test site was located in the cultivation greenhouse of the Wanlvyuan Ecological Breeding Co., Ltd., in Foshan, Guangdong, China, from May to June of 2022. The experiment was carried out by soaking the three culture barrels (60 cm deep and 1.4 m in diameter) and the bottom sand in a barrel with LIRCON 84^®^ disinfectant for 30 min to prevent residual DNA from affecting the subsequent experiment. The culture barrels and bottom sand were then washed to remove residual disinfectant. After ensuring that the sand at the bottom of each bucket was 5 cm thick, and each barrel was filled with tap water such that the depth of water in the bucket was 20 cm above the sand, each barrel was covered with sterile plastic wrap to prevent exogenous pollution. The water temperature was maintained at 27 °C, and the filtration device was opened for aeration. After aeration for 2 days, 2 L of water samples were collected from the center of each barrel, and 2 L of tap water were collected as a blank control in the same room. The same volume of water was added to each barrel after collecting the samples, which were stored in a foam box with ice blocks for preservation and immediately taken back to the laboratory for filtration. The collected water samples were filtered using a 1 μm nylon membrane, followed by DNA extraction using the DNeasy PowerWater Kit for fluorescent qPCR.

The animals (*n* = 18) used in the experiment were the *P. cantorii* artificially bred by the Pearl River Fisheries Research Institute and Wanlvyuan Ecological Breeding Co., Ltd., in 2021. Cultured Asian large softshell turtles were kept in greenhouses at 27 °C, where the water body was constantly filtered, and were fed mosquito fish (*Gambusia affinis*) daily. A total of 81-year-old turtles were selected randomly from the 1-year-old artificial feeding barrel, and six turtles were put into each barrel after weighing. The average weight of the *P. cantorii* in each barrel was controlled as the same (average 204.43 ± 19.85 g for barrel 1, 204.74 ± 13.49 g for barrel 2, and 204.31 ± 27.48 g for barrel 3) on day 0. Simultaneously, approximately 30 *Gambusia affinis* were placed in live baits and added according to turtle feeding conditions.

After the *P. cantorii* were introduced, 2 L water samples (sample, 1 d) were collected from each of the three culture barrels, 2 L tap water samples were collected as negative controls, and the same volume of tap water was added to the culture drums. The water samples were stored in a foam box with ice cubes on days 1, 2, 3, 4, 5 and 6. After transferring to the laboratory, the samples were filtered using a 1 μm nylon membrane. DNA was extracted for qPCR using the DNeasy PowerWater. GraphPad Prism software (version 8.0) was used to analyze daily cycle threshold (Cq) values. When no significant difference in Cq value was found, no more water samples were collected, and the *P. cantorii* were removed and weighed for the next phase of the experiment.

After the Asian giant softshell turtles were removed (day 0), 2 L water samples were collected from each of the three culture barrels, 2 L tap water samples were collected as blank controls, and tap water of the same volume was added to the culture barrels on days 2, 4, 6, 8, 9, and 10 (on day 8, one sample was negative, so we continued with daily sampling; all samples were not detected on day 10). Water samples were stored in a foam box with ice cubes. After they were returned to the laboratory, they were filtered using a 1 μm nylon membrane. DNA was extracted using a DNeasy PowerWater Kit and subjected to qPCR analysis. The number of days required for eDNA degradation was recorded when the DNA concentration was below the limit of detection (LOD).

### *P. cantorii* eDNA concentration and biomass

To evaluate the relationship between *P. cantorii* biomass and eDNA concentrations, three 1-year-old juvenile *P. cantorii* were randomly selected and weighed (211.72 g for barrel 1, 209.42 g for barrel 2, and 206.59 g for barrel 3), one turtle was placed into each culture barrel, and an appropriate amount of mosquito fish was provided as live bait. According to the turtle feeding behavior, mosquito fish were subsequently supplemented (day 0). Following the above steps, experiments with biomass of two turtles on day 3 (average 199.03 ± 17.95 g for barrel 1, 197.97 ± 16.19 g for barrel 2, and 197.61 ± 12.70 g for barrel 3), four turtles on day 6 (average 209.14 ± 24.80 g for barrel 1, 208.23 ± 20.77 g for barrel 2, and 210.07 ± 23.66 g for barrel 3), and eight turtles on day 9 (average 210.59 ± 24.66 g for barrel 1, 210.56 ± 16.69 g for barrel 2, and 211.34 ± 21.60 g for barrel 3) continued. A total of 2 L water samples from each of the three culture barrels were collected each day from day 0 to day 12, 2 L of tap water samples were collected as blank controls, and the same volume of tap water was added to the culture barrels. Water samples were stored in a foam box with ice cubes. After returning to the laboratory approximately 1 h later, the samples were filtered using a 1 μm nylon membrane, and DNA was extracted using the DNeasy PowerWater Kit for qPCR.

### qPCR and data analysis

The ND5 gene fragments of *P. cantorii* in seven different distribution regions were compared by DNAMAN software. All experiments were performed using the QuantStudio 6 qPCR instrument. After quantification, QuantStudioTM Real-Time PCR Software v1.2 was used for preliminary data processing. To avoid contamination, the filtration, DNA extraction and qPCR set-up were performed in three separate rooms. Half an hour before each experiment, nucleic acid eliminator (Vazyme^®^, Nanjing, China) was sprayed to remove residual nucleic acids from the room. For each test, we ran a standard curve, repeated each sample three times, repeated qPCR three times for each repetition, and used three wells of no-template ddH_2_O as negative control for each qPCR. All the qPCR reactions were set for 40 cycles. No amplification was observed for negative or blank controls.

The weight data of *P. cantorii*, Cq values at different annealing temperatures, and different primer probe concentrations were processed and analyzed using Excel software and expressed and compared using mean and standard deviation. Data for standard curves, stability, degradation times of eDNA, and biomass *vs* eDNA concentration were analyzed and plotted using GraphPad Prism software (version 8.0). The standard curve and the relationship between the biomass and concentration of *P. cantorii* eDNA were analyzed and plotted using the linear regression method. The stable time of *P. cantorii* eDNA concentration were analyzed and graphed by ordinary one-way ANOVA. The eDNA degradation time diagram for *P. cantorii* was plotted by nonlinear regression method.

### Ethics statement

The animals used in this research passed the ethical review by the Laboratory Animal Ethics Committee Pearl River Fisheries Research Institute, Chinese Academy of Fishery Sciences. No. LAEC-PRFRI-2022-04-88. The Asian giant softshell turtles used in the experiment did not suffer from any damage.

## Results

### ND5 gene fragments in *P. cantorii* from seven different regions

**Figure 2:**
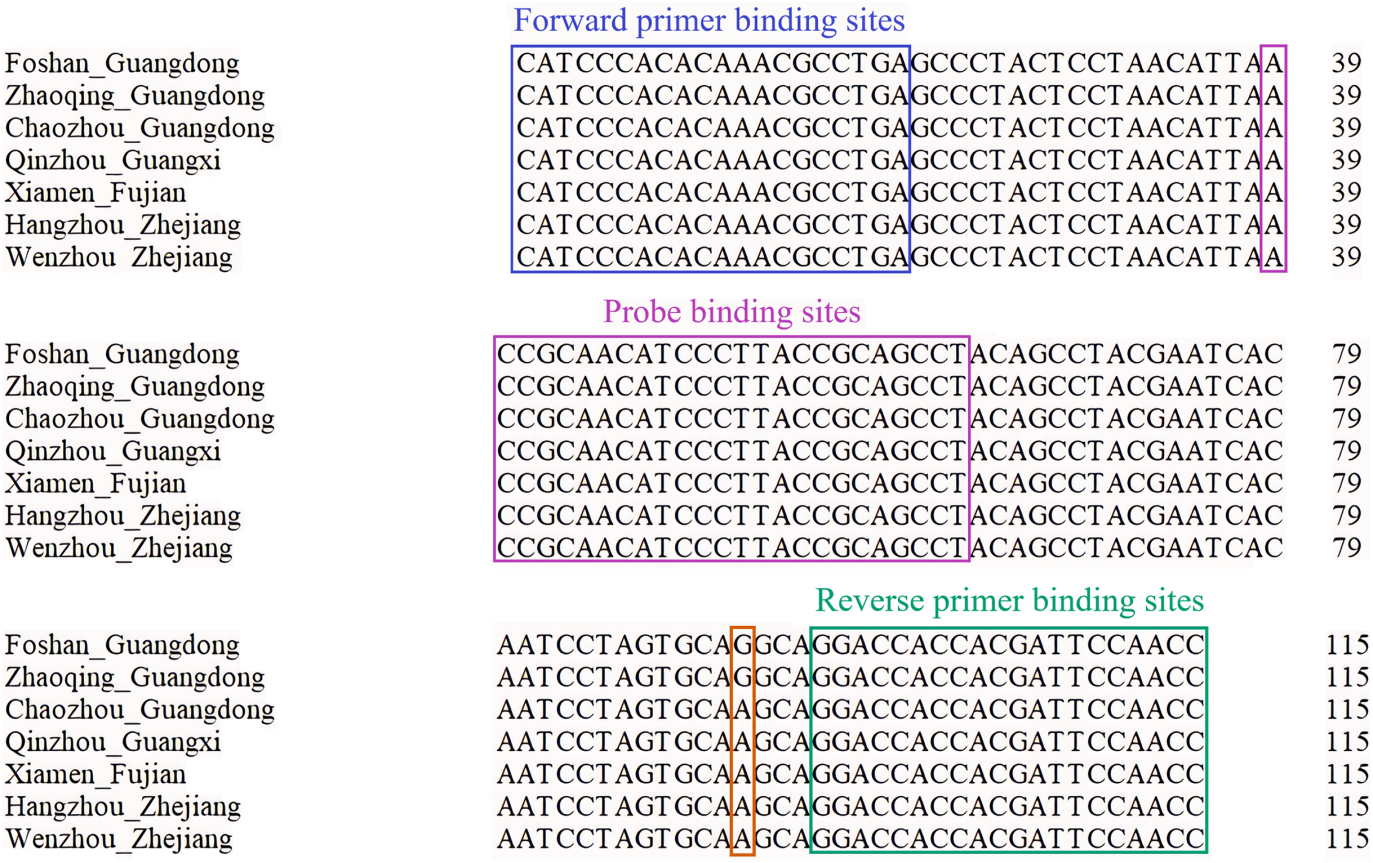
Comparison of the amplified *ND5* fragments of Asian giant softshell turtle (*Pelochelys cantorii*) from seven regions.

After comparing the target gene sequences amplified using the *ND5* primers from the seven regions, namely Foshan, Zhaoqing, Chaozhou, Qinzhou, Xiamen, Hangzhou, and Wenzhou, we found that the target gene sequences of the *P. cantorii* in Foshan and Zhaoqing were the same. Those in the other five regions were the same with only one base difference, indicating that the designed primer could amplify DNA from *P. cantorii* from different regions (Fig. 2).

**Figure 3:**
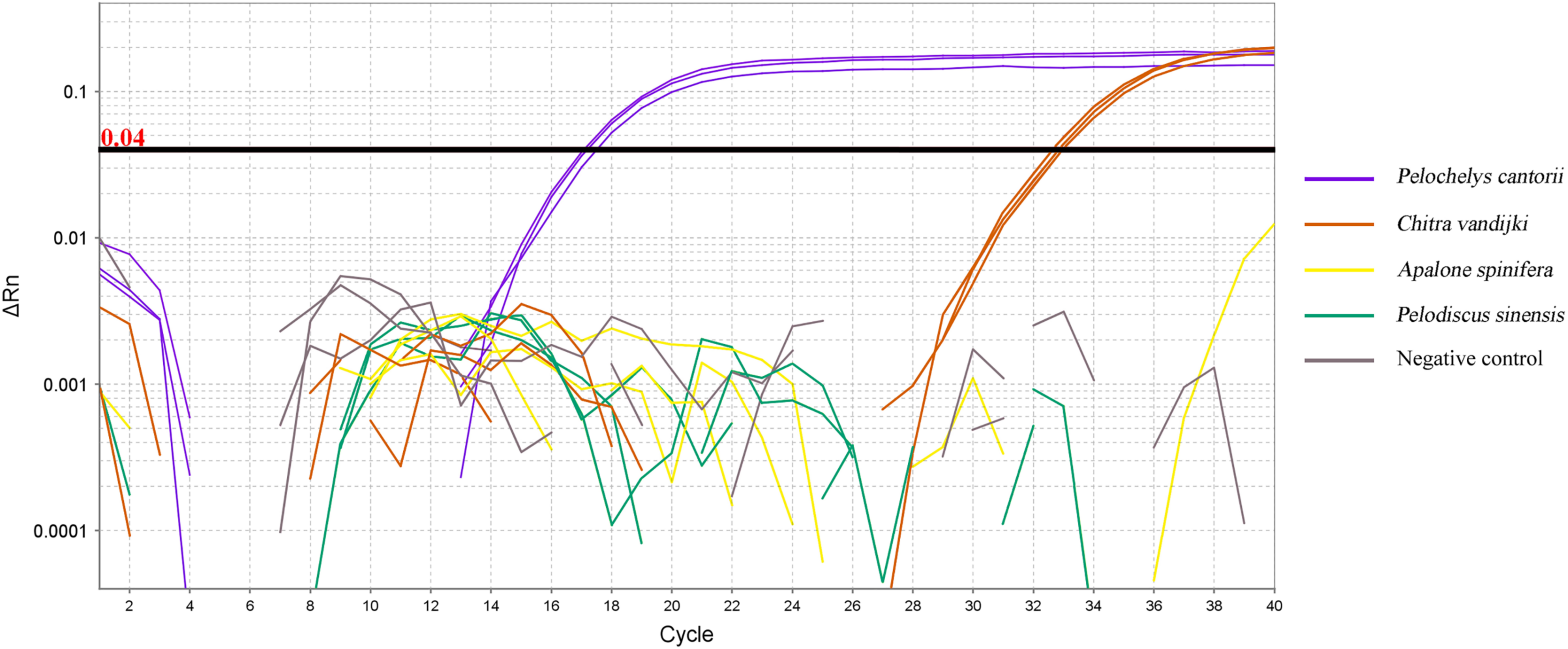
qPCR amplification curve of *ND5* primer and probe pairs for four species.

The DNA of the Asian giant, Chinese, spiny, and Burmese narrow-headed softshell turtles were amplified using qPCR. The DNA of *P. cantorii* and *C. vandijki* were positively amplified, while those of the *P. sinensis* turtle and *A. spinifera* did not amplify (Fig. 3).

### Experimental conditions for qPCR detection of *P. cantorii* eDNA

After qPCR amplification of *P. cantorii* tissue DNA with the same concentration using three different annealing temperatures, the Cq value was found to be the smallest (16.28 ± 0.11) at an annealing temperature of 60 °C, which is the same as the annealing temperature given by Primer Express 3.0 for forward and reverse primers and TaqMan probe. A significant difference between the Cq value at 60 °C and at 63 °C (*p* = 0.0032), a significant difference between the Cq values at 63 °C and 65 °C (*p* = 0.0417), and no significant difference between the Cq values at 60°C and 65°C was found, as shown in Table 3. Based on a comprehensive analysis of significant differences and a comparison of average Cq values, 60 °C was determined as the best annealing temperature for designed primers/probe among the three annealing temperatures.

**Table 3:** Relationship between annealing temperature and daily cycle threshold (Cq) value.

Annealing temperature	Cq-value
60 °C	16.28 ± 0.11
63 °C	17.02 ± 0.17
65 °C	16.48 ± 0.27

**Figure 4:**
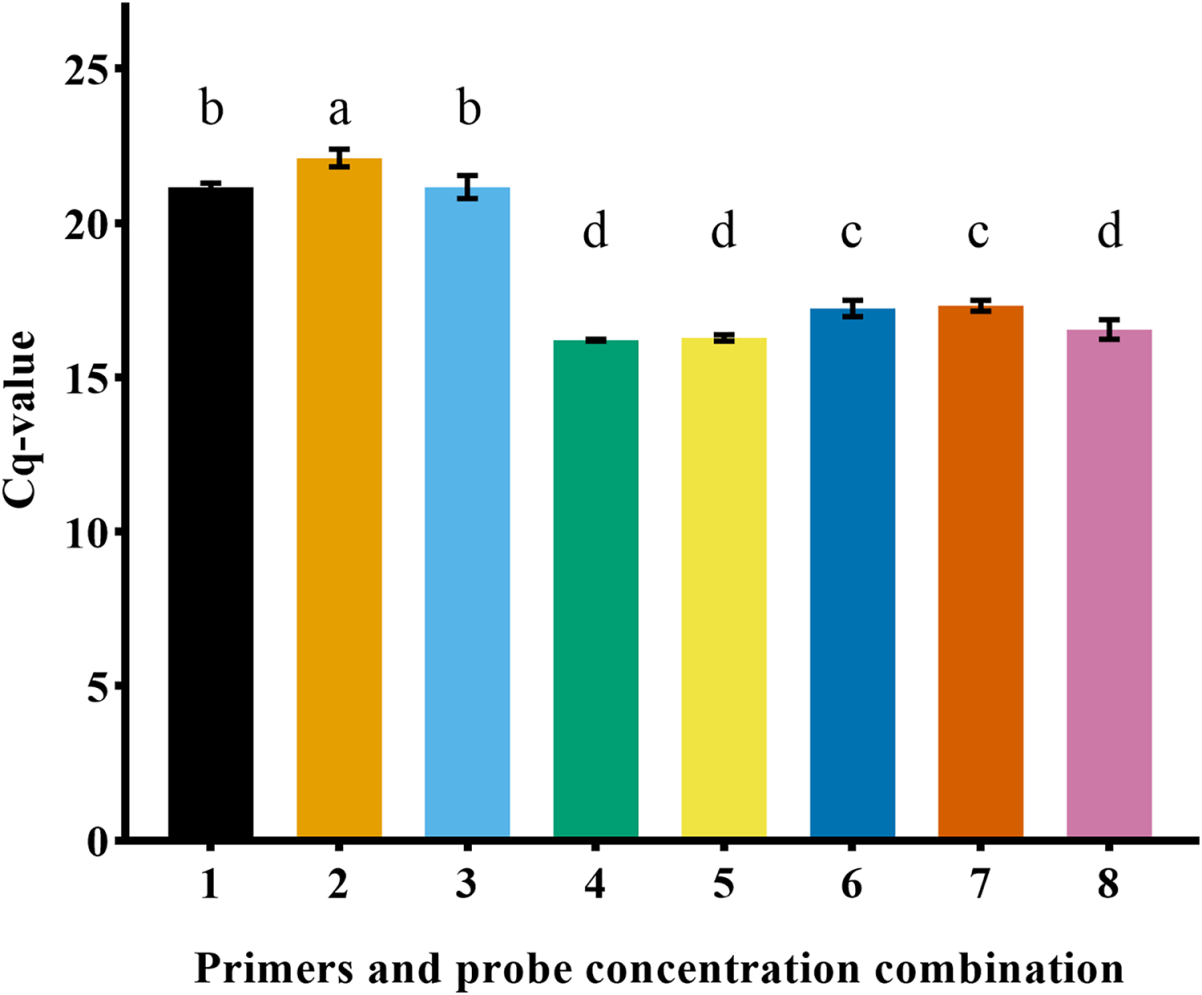
Comparison of Cq value of primer probe combination with different concentrations. Different lowercase letters represented significant difference (*P* < 0.05).

In this experiment, the combinations of different primer and probe concentrations were tested, and the Cq value obtained under the conditions of positive and negative primer (1 μM) and probe (1 μM) were the smallest (16.20 ± 0.03). Thus, the optimal primer-probe concentration combination was identified (Fig. 4).

### Standard curve

**Figure 5:**
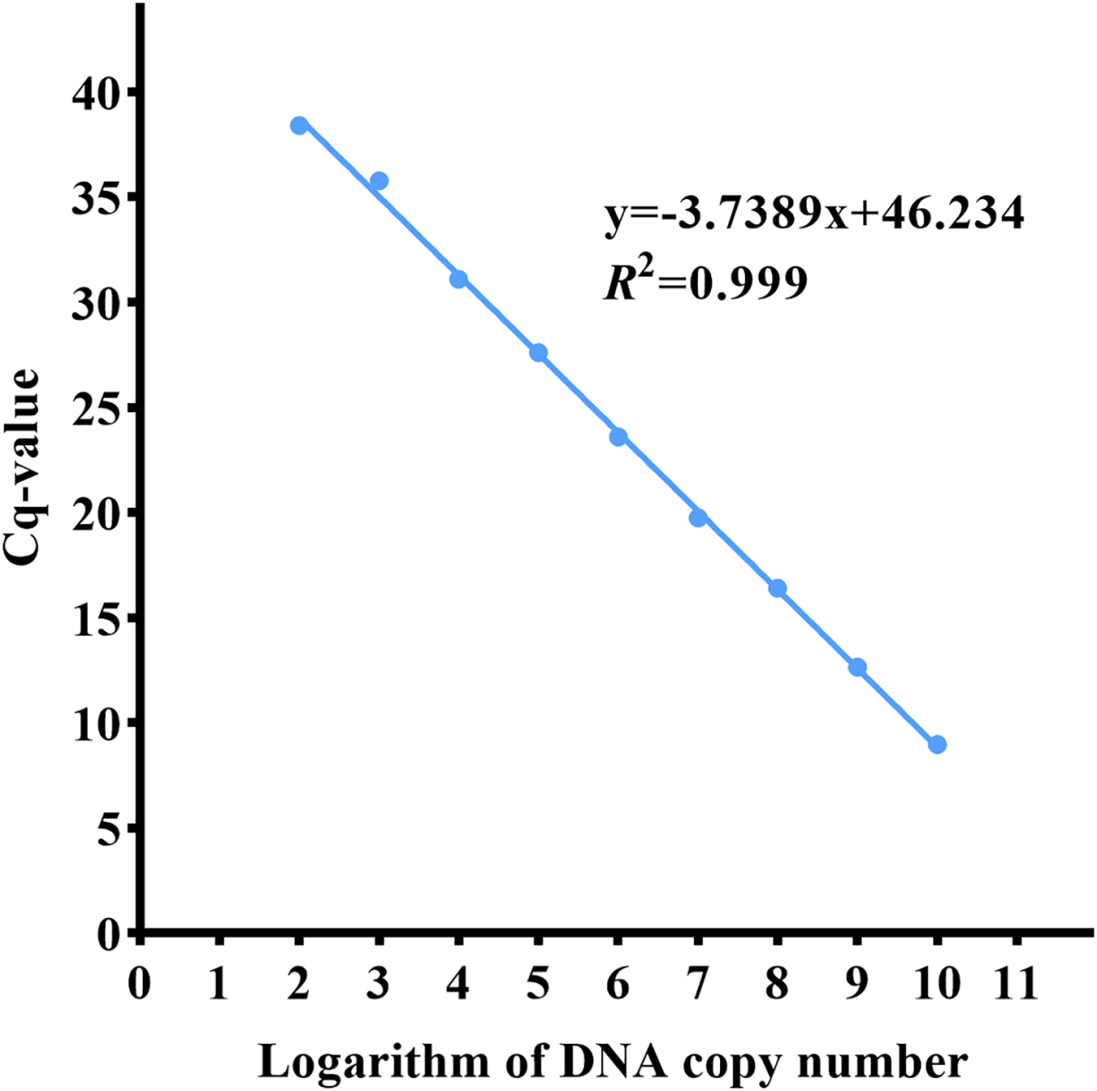
qPCR standard curve of the Asian giant softshell turtle (*Pelochelys cantorii*) *ND5* gene.

The qPCR experiment was carried out on the control substances with a copy number of 10^1^–10^10^ copies/µL. The detection threshold of the *ND5* primer probe was 10^2^ copies/µL, and the standard curve equation was y = −3.7389x + 46.234, r ^2^ = 0.999 (Fig. 5).

### *P. cantorii* eDNA concentration stability and degradation days

**Figure 6:**
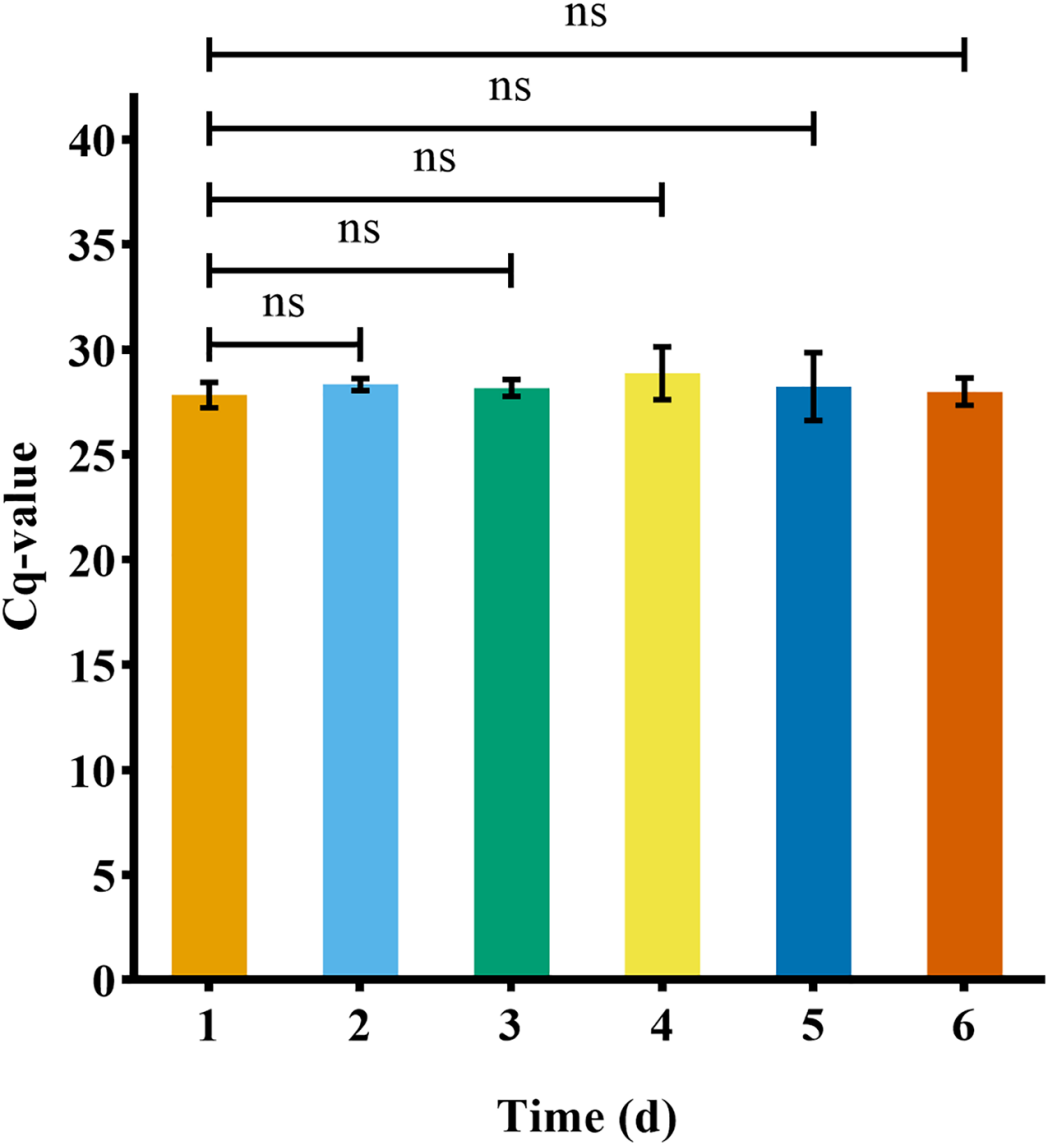
eDNA concentration stability of Asian giant softshell turtle (*Pelochelys cantorii*) over time.

**Figure 7:**
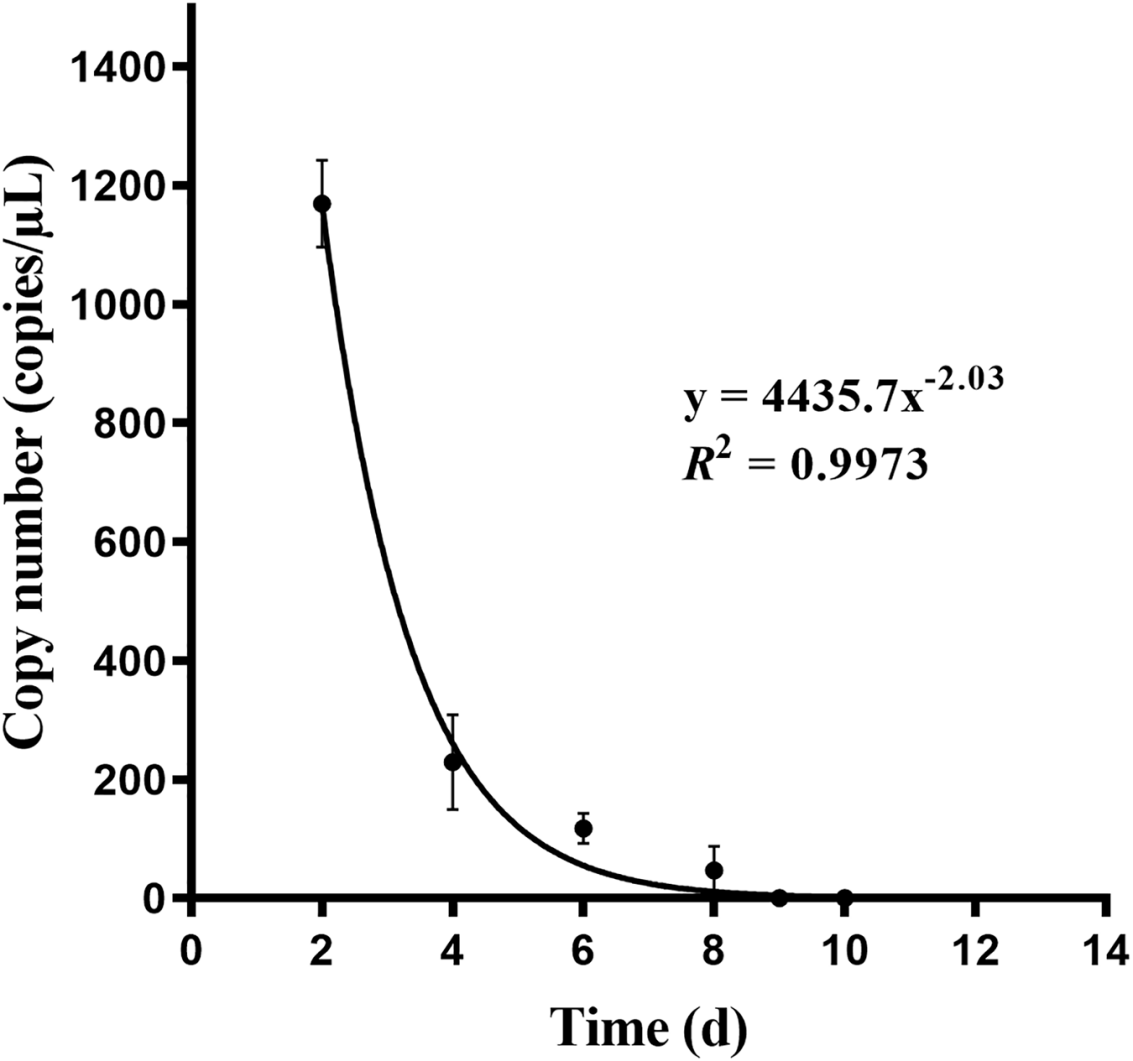
Relationship fitting of eDNA degradation of Asian giant softshell turtle (*Pelochelys cantorii*) over time.

The eDNA reached a stable state after 1 day in water (*n* = 3) (Fig. 6). After removing the turtle, the number of turtle eDNA copies per uL of DNA extract detected in the three experimental buckets within 24 h (day 1) was 1,168.71 ± 141.31 copies/μL (*n* = 3). On day 8, the amplification result of one bucket was negative, and the average eDNA concentration of the other two buckets was 69.64 ± 30.74 copies/μL (*n* = 2). On the 9th day, the results of all three buckets were negative. The amplification results for the negative controls in each experiment were negative (Fig. 7).

### Relationship between eDNA concentration and biomass in *P. cantorii*

**Figure 8:**
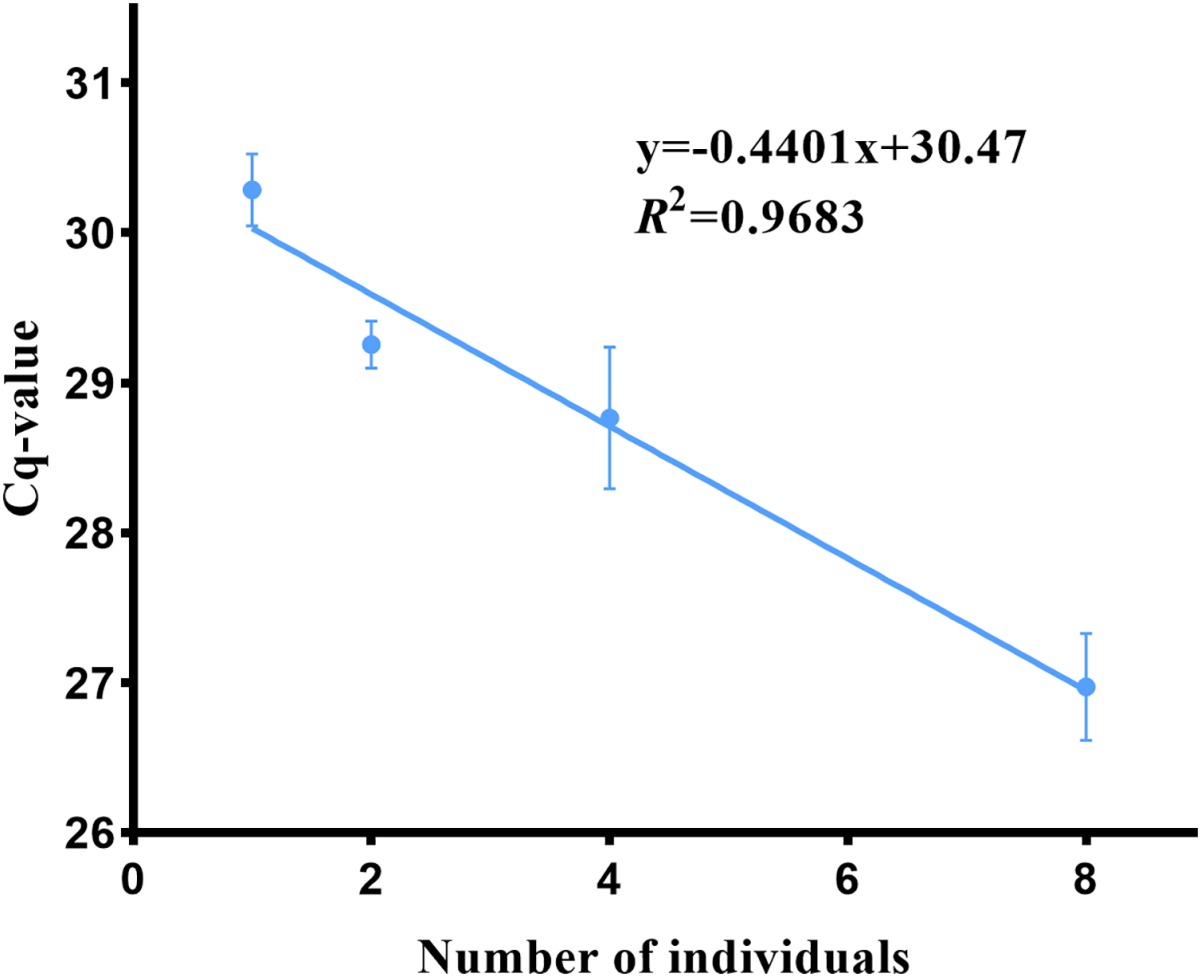
eDNA concentration and biomass of Asian giant softshell turtle (*Pelochelys cantorii*).

The biomass of Asian giant softshell turtles was determined based on the relationship between turtle biomass and eDNA concentration (Cq value). The Cq values in turtle breeding barrels 1, 2, 4, and 8 were 30.29 ± 1.96, 29.25 ± 0.63, 28.78 ± 0.47 and 26.97 ± 0.69, respectively. The correlation curve between *P. cantorii* DNA concentration and the individual number was obtained as follows: y = −0.4401x + 30.47 (Fig. 8); a lower Cq value indicates a higher DNA concentration. Amplification was not detected in the negative controls. There was a positive correlation (R^2^ = 0.968, *p* = 0.0160, 95% confidence interval) between the target DNA concentration and the number of *P. cantorii*.

## Discussion

This study demonstrated that the designed *ND5* primers and TaqMan probe amplified the DNA of *P. cantorii* and *C. vandijki*, but the amplification efficiency of *C. vandijki* was lower than that of *P. cantorii*, where other turtle species and the blank control did not produce amplification signals. *C. vandijki* cannot be found in natural ecosystems in China (Platt et al., 2014), so the designed primers and probe could be applied for eDNA detection in *P. cantorii*. Becaue the eDNA concentration in endangered aquatic animals is low in riverine ecosystems, which have a certain fluidity, detection is difficult and a more efficient detection technique is required. Compared to conventional PCR, the TaqMan probe method for real-time fluorescent qPCR detection can improve detection accuracy and sensitivity, and quantitative analysis can further obtain real-time eDNA concentrations (Takahara et al., 2012). As the eDNA concentration is related to population biomass, its trend can reflect the relative biomass dynamics of endangered species (Nguyen et al., 2016; Mizumoto et al., 2018; Yang et al., 2020; Yin et al., 2021; Yan et al., 2022). In this study, the TaqMan probe was used in conjunction with qPCR. The results showed a strong linear relationship between the threshold cycle number of qPCR and the concentration of the standard substance over a wide linear range. These results indicate that under laboratory conditions, the residual DNA of Asian giant softshell turtles could be specifically and quantitatively detected after effective extraction from the experimental water environment.

In the present study, we identified a linear correlation between the eDNA concentration of *P. cantorii* and biomass (R^2^ = 0.968). The correlation between eDNA concentration, density and biomass of aquatic species has also been observed in other studies (Takahara et al., 2012; Mizumoto et al., 2018; Li et al., 2019; Muri et al., 2020; Yan et al., 2022), indicating that a further refined method can estimate the relative abundance of species from eDNA and provide a powerful tool for species protection and management. However, the relationship between species biomass and eDNA concentration is not always consistent because of complex water flow, habitat conditions, and other factors (Doi et al., 2017; Ghosal et al., 2018; Coulter et al., 2019). Coulter et al. (2019) found a positive and nonlinear relationship between silver carp (*Hypophthalmichthys molitrix*) biomass density and eDNA concentration and detection rate. The eDNA concentration and detection rate increased rapidly with an increase in silver carp density, but tended to stabilize at medium density. Species-specific testing suggests that designing and developing eDNA testing methods is feasible for the *P. cantorii*. This will provide information relevant for its conservation, describe its distribution and abundance in the natural range of *P. cantorii* and other rivers, and allow regular monitoring to study population changes over time and any anthropogenic impacts on the habitat. Successful detection of eDNA in laboratory samples does not guarantee success in aquatic habitats, where eDNA concentrations are likely to be much lower (Coulter et al., 2019) and can be affected by factors such as the abundance of the target species, water flow rate, water temperature, UV radiation, pH and some inhibitors like humic acid and humus (Davy, Kidd & Wilson, 2015). Although Davy, Kidd & Wilson (2015) successfully detected nine species of turtles in the wild using eDNA, they strongly recommend considering various environmental factors. Therefore, in future applications in natural environment, natural environment factors should be considered and optimizations such as increasing the number of PCR cycles (Wilcox et al., 2013) and diluting DNA samples (Thomsen et al., 2012) to balance sensitivity and accuracy should be implemented.

After *P. cantorii* was removed from the water, the eDNA concentration in the water body correlated negatively with time, and its retention time in the water body was 9 days. The lifetime of eDNA in water ranges from 1 day to a few weeks, depending on a variety of environmental factors, such as temperature, pH, and propagation distance, whereas the effects of ultraviolet light have been varied or even been contradictory in previous studies (Pilliod et al., 2014; Strickler, Fremier & Goldberg, 2015; Deiner & Altermatt, 2017; Tsuji, Yamanaka & Minamoto, 2017; Mchler, Osathanunkul & Altermatt, 2018). In addition, the eDNA degradation rate is higher in environments with higher species biomass density (Bylemans et al., 2018). These non-biological and biological factors contribute to increased microbial activity and abundance in water, thus indirectly affecting eDNA degradation (Strickler, Fremier & Goldberg, 2015). Studying organism-specific eDNA degradation is important for the monitoring of rare and endangered species. A study on Rock carp (*Procypris rabaudi*) showed that the eDNA copy number correlated negatively with time after removal of *P. rabaudi*, and its retention time in water was 17 days (Yan et al., 2022). In this study, the retention time of *P. cantorii* eDNA was 9 days.

Aquatic wildlife resource surveys typically use nets that are relatively reliable for monitoring high-abundance species. However, the capture probability of low-abundance and endangered species is low. As wildlife resources continue to decrease, the results of traditional resource surveys may become inaccurate (Magnuson, Benson & McLain, 1994). The environmental DNA monitoring technology established in this study can also be used to monitor the DNA of *P. cantorii* in natural water bodies, thereby reducing the scope of traditional resource surveys and manpower. This may also increase monitoring accuracy.

## Conclusions

In this study, a TaqMan qPCR assay was designed and screened for the *ND5* gene of the *P. cantorii* mitochondrial genome. The relationship between the biomass of *P. cantorii* and eDNA concentration was explored in a laboratory environment, and the eDNA concentrations of different numbers of *P. cantorii* cultured in the laboratory were determined. Although the distribution of *P. cantorii* in natural ecosystems in China is very rare, testing the application of the optimized qPCR monitoring system to samples from natural ecosystems is necessary. However, rapid identification of locations where this endangered species may exist in the natural environment can help prioritize habitat protection for this species, while reducing the use of human resources for surveys and capture for artificial breeding purposes.

## Supplemental Information

10.7717/peerj.16218/supp-1 Supplemental Information 1 CT values of qPCR experiments with eDNA water samples of different numbers of Asian softshell turtles. The relationship between the number of individuals and CT value and the relationship between eDNA degradation days and CT value. The mitochondrial genome sequences of Asian softshell turtles from Zhaoqing, Chaozhou, Qinzhou and Hangzhou.

10.7717/peerj.16218/supp-2 Supplemental Information 2 Author Checklist.

## Additional Information and Declarations

### Data Availability

The following information was supplied regarding data availability: The raw data is available in the Supplemental Files.
